# Effectiveness of Intra-oral Non-skeletally Anchored Appliances in the Correction of Skeletal Class III Malocclusion in Growing Patients: A Systematic Review and Meta-Analysis of Randomized Controlled Trials

**DOI:** 10.7759/cureus.110262

**Published:** 2026-06-04

**Authors:** Yazan M. Kourbaj, Mohammad Y Hajeer, Jehad M. Kara-Boulad, Ahmad S. Zakaria, Jihad Nouman Abou Nassar, Doa'a Tahseen Alfailany, Mohammad Khursheed Alam

**Affiliations:** 1 Department of Orthodontics, Faculty of Dentistry, University of Damascus, Damascus, SYR; 2 Department of Orthodontics, Faculty of Dentistry, Mari Private University, Idlib, SYR; 3 Department of Orthodontics, School of Dental Sciences, Universiti Sains Malaysia, Kota Bharu, MYS; 4 Department of Fixed Prosthodontics, Faculty of Dentistry, University of Damascus, Damascus, SYR; 5 Department of Preventive Dental Science, College of Dentistry, Jouf University, Sakaka, SAU

**Keywords:** angle class iii malocclusion, class iii correction, class iii treatment, early class iii treatment, growing patients, lower-clear-plate based intermaxillary traction, mixed dentition, non-skeletally-anchored appliances, orthodontic appliances, removable mandibular retractor

## Abstract

Extra-oral appliances used for correcting skeletal Class III malocclusion are often bulky, unaesthetic, and heavily reliant on patient compliance, which can negatively affect treatment outcomes, particularly in growing patients. As a result, intra-oral alternatives have gained increasing attention due to their improved comfort, better acceptance, and reduced visibility. The objective of this systematic review was to examine and appraise existing evidence concerning the effectiveness of intra-oral, non-skeletally anchored appliances for correcting skeletal Class III malocclusion in growing individuals. A comprehensive and systematic search was conducted across five major electronic bibliographic databases, with no language restrictions, to ensure the broad inclusion of relevant studies. Only randomized controlled clinical trials (RCTs) assessing intra-oral non-skeletally anchored appliances were included in the review.

A total of eight RCTs involving 336 growing patients met the inclusion criteria, although only two studies were suitable for quantitative meta-analysis. Among the evaluated appliances, the removable mandibular retractor (RMR) demonstrated forward movement of key skeletal landmarks, including point A (mean difference (MD) = 1.92 mm), point B (MD = 0.13 mm), prosthion (Pr) (MD = 1.97 mm), and infradental (Id) (MD = 0.16 mm). Additionally, RMR use was associated with a significant increase in the facial convexity angle (MD = 4.92°) and a reduction in the nasolabial angle (MD = 5.18°), indicating favorable soft tissue profile changes. The lower-clear-plate-based intermaxillary traction (LCP-IMT) appliance demonstrated the most significant improvement in sagittal skeletal relationship. It showed the greatest increase in the *ANB angle*, the largest reduction in the *SNB angle*, and the smallest increase in mandibular length, suggesting effective restriction of mandibular growth. Overjet increased significantly with all evaluated appliances, whereas overbite increased only with LCP-IMT. A consistent decrease in lower incisor inclination was observed across all appliances. Soft-tissue adaptations across the appliances included increased facial convexity, forward positioning of the upper lip, and posterior repositioning of the lower lip, all of which contributed to improved facial esthetics.

In conclusion, LCP-IMT and reverse twin block (RTB) appliances demonstrated the most favorable skeletal improvements in the sagittal dimension. These were followed by the modified RMR, conventional RMR, modified tandem appliance (MTA), and Frankel’s function regulator III. However, the overall strength of evidence was between low and very low due to methodological limitations, small sample sizes, and heterogeneity among the included studies, highlighting the need for further high-quality research.

## Introduction and background

Managing skeletal Class III malocclusion is a significant challenge in contemporary orthodontic practice [1]. It is crucial to identify whether the etiology of the Class III malocclusion is skeletal, dental, or functional [2]. Skeletal Class III is a condition where there is an imbalance in the relationship between the upper and lower jaws, often resulting in maxillary retrusion, mandibular protrusion, or both [3], while dental Class III can occur due to protrusion of the lower incisors and retrusion of the upper incisors [4]. Pseudo-functional Class III malocclusion, a condition in which the lower jaw shifts forward during biting, is characterized by a forward slide of the mandible from the rest position to maximal intercuspation. Diagnosis involves assessing the path of closure [5].

Early intervention for Class III malocclusion is commonly recommended in the literature because it may enhance growth-related outcomes and lead to more favorable occlusal relationships during the development stage [2]. Functional treatment for growing patients with skeletal Class III aims to apply growth modification to stimulate the maxillary growth and restrict the mandibular development [6]. Growth modification becomes less effective in older patients, and treatment options are limited to camouflage for mild-to-moderate cases. When the skeletal deformity is severe, orthognathic surgery is the only solution [7].

**Figure 1:**
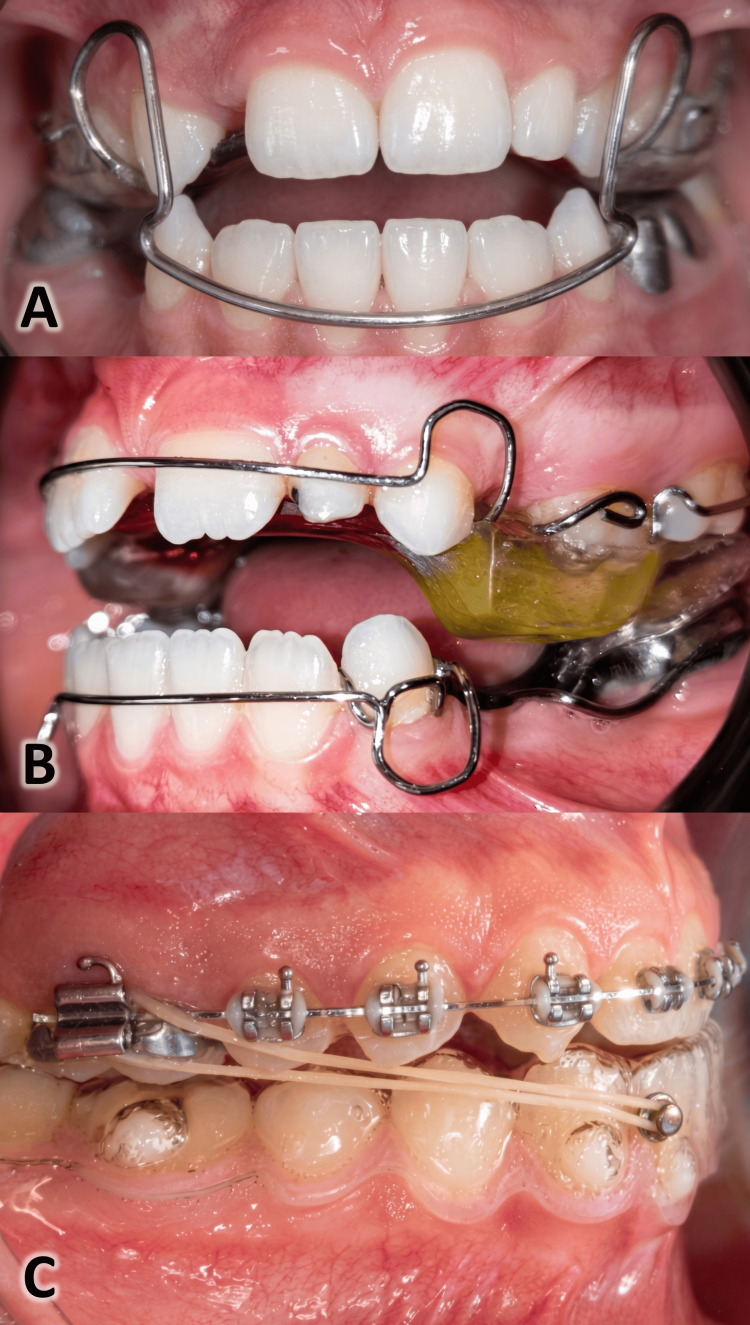
Examples of functional appliances used in the correction of Class III malocclusion in growing patients. A: removable mandibular retractor (RMR). B: reverse twin block (RTB). C: lower-clear-plate-based intermaxillary traction (LCP-IMT) These intra-oral images are taken from patients treated by the first author (YMK) during his residency training.

Growth modification with intra-oral or extra-oral appliances can be an effective therapeutic option for treating skeletal Class III malocclusion in growing patients [8]. Various methods have been employed to guide growth in patients presenting with skeletal Class III malocclusion. Examples include extra-oral appliances like the face mask [9] and the chin cup [10]. ]. Orthodontic intra-oral appliances can generally be divided into two main types. The first includes appliances that use skeletal anchorage (i.e., fixed to bone) to correct Class III malocclusion [11]. The second type includes appliances that apply forces between the upper and lower jaws or use natural muscle activity to influence the teeth and supporting bone [12]. Examples of this group include the Frankel III appliance [13], the removable mandibular retractor (RMR, Figure 1A) [14], the reverse twin block (RTB, Figure 1B) [15], the Activator III [16], the lower-clear-plate-based intermaxillary traction (LCP-IMT, Figure 1C) [6], and the Bionator III [17].

Extra-oral appliances have been shown to be effective in treating Class III patients with skeletal changes, but obtaining patient cooperation can be a major challenge [18]. These appliances, often large and unattractive, may discourage patient compliance. As a result, the successful use of these appliances requires significant patience and dedication, which is not always achievable. On the other hand, intra-oral non-skeletally anchored appliances such as the reversed twin block and removable mandibular retractor tend to achieve better patient compliance [19]. However, skeletally anchored intra-oral appliances can cause significant discomfort, particularly immediately after being placed [20]. Some clinical studies have been conducted on the effectiveness of intra-oral, non-skeletally anchored appliances in correcting Class III skeletal malocclusion. However, conflicting results have been reported in these studies. For instance, Saleh et al. reported that point B moved forward by a mean of 0.17 mm when treating 6- to 9-year-old children using the RMR appliance [2], whereas Baccetti and Tollaro reported a mean of 0.47 mm backward movement of point B using the same appliance on a comparable treatment group of children [21].

While reviewing the literature, several systematic reviews have evaluated the evidence on the effectiveness of available tools for correcting Class III malocclusion in growing patients [22,23]. The systematic review by Owens et al. [22] included both extra-oral and intra-oral orthodontic appliances, with and without skeletal anchorage, for the management of Class III malocclusion in children and adolescents, covering a broad age range extending into late adolescence (approximately 16 years), which is generally considered beyond the optimal window for many early orthopedic interventions. This wide age span (5-16 years) combines patients at different growth stages, and the high heterogeneity between the studies may further reduce the reliability of pooled estimates and limit their direct clinical applicability.

Similarly, the systematic review and meta-analysis by Alhamwi et al. [23] evaluated a range of extra-oral and intra-oral orthodontic appliances in growing patients; however, its analysis was primarily limited to soft-tissue outcomes. Furthermore, the review combined two categories of appliances with substantially different biomechanical characteristics and treatment principles. These appliances differ considerably in the degree of patient compliance required, as well as in their mechanisms of action and their effects on skeletal and dentoalveolar structures. Therefore, combining such fundamentally different treatment modalities within a single review may limit the clinical relevance and interpretability of the findings. Most systematic reviews have primarily examined extra-oral appliances like the face mask [24] and chin cup [25].

Additionally, numerous systematic reviews were conducted to evaluate the effectiveness of skeletally-anchored intra-oral appliances in correcting Class III malocclusion in growing patients [26,27]. A preliminary search of recent systematic reviews and clinical studies was conducted to identify existing evidence on Class III malocclusion management in growing patients. However, no systematic review has specifically evaluated intra-oral non-skeletally anchored appliances for correcting Class III malocclusion in growing patients. In other words, a systematic review specifically addressing functional Class III appliances or intra-oral inter-maxillary traction appliances is lacking in the current literature. Therefore, the present study was undertaken to respond to the following targeted research question: "What is the best non-skeletally anchored intra-oral appliance for the management of skeletal Class III malocclusion in growing patients?"

## Review

Materials and methods

Preliminary Search and Protocol Registration 

A preliminary PubMed search was conducted prior to finalizing the systematic review protocol to identify existing reviews and gather relevant literature on the topic. The protocol was registered early in the process in the PROSPERO database (CRD42025633053). This review was developed following the Cochrane Handbook for Systematic Reviews of Interventions [28] and the /Preferred Reporting Items for Systematic Reviews and Meta-Analyses (PRISMA) guidelines [29,30].

Eligibility Criteria

The Population, Intervention, Comparison, Outcomes, and Study (PICOS) framework was used to identify the key components of the review and to establish the inclusion and exclusion criteria for eligible studies. Regarding the targeted “population,” growing patients between 6 and 12 years of age, of any gender or ethnic group, with skeletal Class III malocclusion were included. Concerning the “intervention,” any orthodontic treatment using intra-oral non-skeletally-anchored Class III appliances was involved. The “comparison” group should include patients who received either no orthodontic treatment or orthodontic treatment with an appliance different from that used in the intervention group. The “outcomes” of interest were the amount of increase in the ANB angle on the lateral cephalometric image (considering the improvement in the SNA angle and the decrease in the SNB angle). Additionally, changes in mandible length (Go-Me) and in Wits appraisal were observed.

Study Design

Inclusion was limited to randomized controlled trials (RCTs), with no language or publication-year restrictions.

The following studies were excluded: case reports and case series; non-randomized clinical trials (CCTs); individual viewpoints; editorial perspectives; systematic reviews; studies without a specified sample size; and studies in which the treatment group comprised fewer than 10 participants.

Search Strategy

An electronic literature search by two reviewers (YMK, MYH) was conducted independently on the 5th of January 2025 across the following databases, with no date or language restrictions: PubMed®, the Cochrane Library, Scopus®, Web of Science™, and Google Scholar™. The keywords used in the electronic search are listed in Table 1, and the complete search methodology is outlined in Appendix 1. Reference lists of the included articles and pertinent review papers were screened to identify additional studies not captured by the electronic search. Additionally, ClinicalTrials.gov and the World Health Organization’s International Clinical Trials Registry Platform (ICTRP) were consulted to locate unpublished studies or ongoing research projects.

**Table 1:** Keywords used in the electronic search

Component of the search strategy	Keywords and related terms
Type of malocclusion	Mixed dentition, Class III malocclusion, skeletal Class III, mesial occlusion, mandibular prognathism, anterior crossbite.
Treatment planning	Growth modification, functional treatment, functional orthopedic, inter-maxillary traction, jaw relationship correction, maxillary advancement, mandibular restriction.
Outcomes	The amount of skeletal improvement, mandibular length, overjet, overbite, upper incisor inclination, lower incisor inclination, maxillary dentoalveolar position, mandibular dentoalveolar position, lip profile, facial profile, facial convexity, nasolabial angle, mentolabial angle, upper lip position, lower lip position.
Intervention	removable functional appliance, Frankel regulator III, Bionator III, Activator III, modified tandem appliance, removable mandibular retractor, reverse twin block, inter-maxillary traction appliance.

Study Selection and Data Extraction

To minimize potential citation bias, this systematic review followed a predefined protocol and structured search strategy based on PRISMA guidelines. Literature identification, screening, and study selection were conducted according to predefined inclusion and exclusion criteria, independent of authorship considerations. Multiple databases were searched using comprehensive search terms to capture the broadest possible body of relevant evidence. The selected trials were independently evaluated for inclusion in this review by two reviewers (YMK and MYH). In the event of conflicts, the third reviewer (MKA) was asked to resolve them. The full texts of relevant articles deemed appropriate for inclusion were evaluated after the initial assessment of titles and abstracts. Articles were excluded if they failed to fulfill any of the inclusion requirements. Two authors (YMK and MYH) independently extracted data and study characteristics from the included trials using predefined extraction tables. In case of disagreements, a third author was involved to settle any conflicts. The tables summarizing study characteristics included general information (author, publication year, journal, country), study design, participant details (sample size, age, eligibility criteria), treatment duration, intervention, assessment methods, and reported outcomes.

Risk of Bias of the Collected Studies

The included studies underwent risk-of-bias evaluation with the Cochrane RoB 2.0 tool [31]. The assessment of bias risk within each trial was carried out independently by the authors (YMK & MYH), and their judgments were compared. Any conflicts were settled with input from a third reviewer (MKA). Five domains were assessed for risk of bias-categorized as high, low, or some concerns-including the randomization process (selection bias), deviations from intended interventions, missing outcome data (attrition bias), outcome measurement, and selection of the reported result (reporting bias). The overall risk-of-bias judgment was determined as follows: studies with all domains rated as low risk were considered low risk overall; studies with at least one domain rated as “some concerns” and no domain rated as high risk were considered to have some concern. A high-risk-of-bias judgment would be made if one or more domains were found to be at high risk of bias, or if concerns were present in several domains, significantly reducing confidence in the result. The researchers used the Risk of Bias Visualization (ROBVIS) tool to graphically present their evaluation of bias [32]. Regarding the quality of evidence, the Grading of Recommendations Assessment, Development, and Evaluation (GRADE) guidelines were applied [33].

Synthesis of the Collected Data

Meta-analyses were conducted using Review Manager (RevMan), Version 5.4 (The Cochrane Collaboration, Nordic Cochrane Center, Copenhagen, Denmark). Continuous outcomes were analyzed with a random-effects model using the inverse variance method, with results reported as mean differences and 95% confidence intervals (CIs). Statistical heterogeneity was considered significant at p < 0.05, and the I² statistic was used to assess its extent. Forest plots were created to visually display the results, and the overall quality of evidence was systematically assessed using the GRADE approach.

Results

Study Selection and the Literature Review

**Figure 2:**
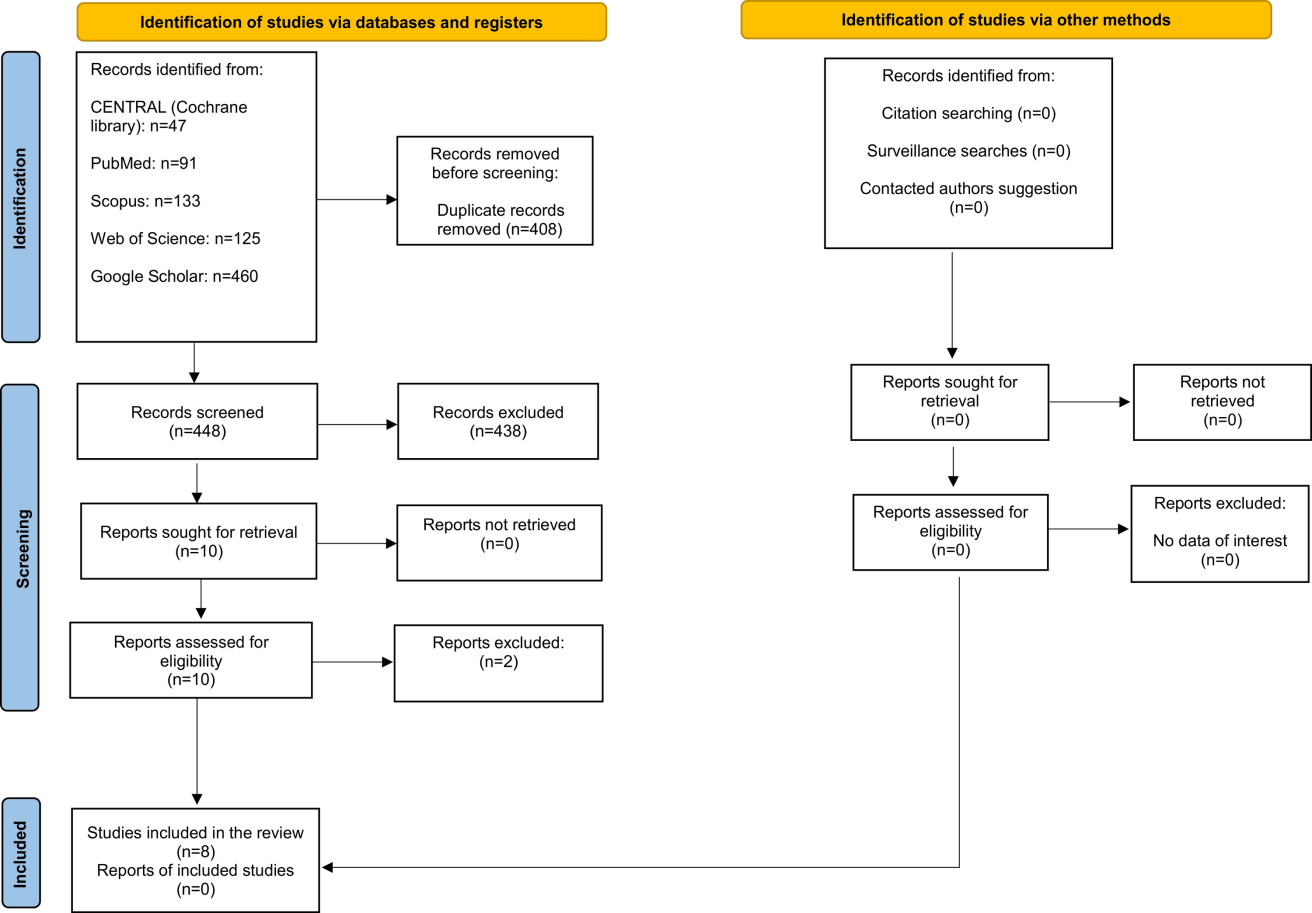
The Preferred Reporting Items for Systematic Reviews and Meta-analyses (PRISMA) flow diagram of the included studies

A total of 856 studies were identified through an electronic search. After removing duplicates, 448 articles were carefully checked. Articles were screened at the title and abstract level to determine eligibility; those not meeting the inclusion criteria were excluded. A full-text review was conducted for 10 studies, with two exclusions. Appendix 2 provides a summary of the excluded articles and the reasons for exclusion. Finally, eight articles [2,6,11,18,19,34-36] were deemed suitable for the systematic review. The Preferred Reporting Items for Systematic Reviews and Meta-Analyses (PRISMA) flow diagram for the review procedure is displayed in Figure 2.

Characteristics of the Included Studies

Table 2 displays the characteristics of the eight included studies. All of the studies were randomized controlled clinical trials [2,6,11,18,19,34-36]. The studies included 336 growing patients. Gender distribution was reported in all studies, with male-to-female ratios varying: six studies reported an approximate 1:1 distribution [2,6,11,18,34,36], 2:1 in one study [19], and 1:1.5 in the last study [35]. All studies reported the ages of the samples, with five studies reporting an age range of 7-9.5 years [2,6,18,34,36] and three studies reporting an age range of 10-11.5 years [11,19,35].

**Table 2:** Characteristics of the included studies m: male, f: female, M-RMR: modified removable mandibular retractor, RMR: removable mandibular retractor, BAIMT: bone anchored intermaxillary traction, RTB: reversed twin block, PBMT: photobiomodulation therapy, RTBLP: reverse twin block with lip pads, FR: Frankel, FM: face mask, MTA: modified tandem appliance, ORTA: orthodontic removable traction appliance, SNA: angle between the anterior cranial base and NA plane, SNB: angle between the anterior cranial base and NB plane, ANB: SNA minus SNB, Co: condylion, Gn: gnathion, TV: T Vertical, Wits: distance between AO-BO, U1-SN: angle between the anterior cranial base and the upper incisor axis, L1-Go-Me: angle between the mandibular plane and the lower incisor axis, Pr: prosthion, Id: infradental, gla.sn.pog: facial convexity angle, Ls-Esth: distance between the Labrale superius and E-Line of Ricketts, Li-Esth: distance between the Labrale inferius and E-Line of Ricketts, NasoLab: nasolabial angle, MentoLab: mentolabial angle, AO-BO: distance measured between perpendicular projections of Point A (AO) and Point B (BO) onto the occlusal plane, TMJ: temporomandibular joint, RME: rapid maxillary expansion, A-TV: horizontal distance between point A and the T vertical line, B-TV: horizontal distance between point B and the T vertical line.

Authors	Number of patients, M/F	Mean age	Intervention	Treatment duration	Outcomes
Ulgen and Firatli, 1994 [36]	Intervention group: 20 (10 m, 10 f) Control group: 20 (10 m, 10 f)	9.5 years 9.3 years	Frankel’s III function regulator, Untreated	The mean treatment period of the FR-3 group was 1.9 years, and the mean observation period of the control group was 1.8 years.	SNA, SNB, ANB, Overjet, Overbite, U1-SN, L1-GoMe
Saleh et al., 2013 [2]	Intervention group: 33 (17 m, 16 f) Control group: 34 (15 m, 19 f)	7.5 ± 1.33 years 7.3 ± 1.58 years	RMR, Untreated	14.5 ± 0.1 months	A-TV, B-TV, Id-TV, Pr-TV, Nasolab, Mentolab, gla.sn.pog
Husson et al., 2016 [18]	Group 1: 16 (8 m, 8 f) Group 2: 16 (7 m, 9 f)	7.98 ± 0.68 years 8.11 ± 0.76 years	MTA, FM	MTA: 7.07 ± 0.78 months. FM: 6.4 ± 1.30 months.	SNA, SNB, ANB, A-TV, Co-Gn, Overjet, Overbite
Majanni and Hajeer, 2016 [11]	Intervention group: 19 (10 m, 9 f) Control group: 19 (11 m, 8 f)	11.3 years 11.5 years	BAIMT, RMR	12 months	SNA, SNB, ANB, Co-Gn, Overjet, Overbite, U1-SN, L1-Go-Me, Nasolab Mentolab, gla.sn.pog, Ls-Esth, Li-Esth
Minase et al., 2019 [35]	Group 1: 13 (3 m, 10 f) Group 2: 13 (6 m, 7 f) Group 3: 13 (6 m, 7 f)	10 ± 3.8 years 10.2 ± 3.7 years 10.3 ± 3.6 years	RTBLP-RME, FM-RME, Untreated	9 months of treatment. The observation period for the control group was also 9 months.	SNA, SNB, ANB, Wits appraisal, Co-Gn, A-TV, U1-SN, L1-Go-Me
Alzabibi et al., 2021 [6]	Intervention group: 21 (12 m, 9 f) Control group: 19 (8 m, 11 f)	8.95 ± 0.88 years 9.14 ± 0.80 years	ORTA, Untreated	Intervention group: 4.34 ± 2.02 months. The observation period for the control group was 6 months.	SNA, SNB, ANB, Wits appraisal, Co-Gn, U1-SN, Overjet, Overbite, Nasolab Mentolab, Ls-Esth, Li-Esth
Khwanda et al., 2022 [19]	Intervention group: 20 (12 m, 8 f) Control group: 20 (14 m, 6 f)	10.27± 0.80 years 10.12 ± 0.84 years	RTB+PBMT, RTB	Until getting an adequate overjet of 2 mm.	3D changes in the TMJ region and the skeletal changes following Class III treatment (SNA, SNB, ANB)
Al Hammadi and Haddad, 2023 [34]	Group 1: 20 (12 m, 8 f) Group2: 20 (9 m, 11 f)	8 ± 0.6 years 8 ± 0.5 years	M-RMR, RMR	Seven months or when positive incisor overjet was achieved	SNA, SNB, ANB, A-TV, B-TV, Overjet, U1-SN, L1-GoMe, Nasolab, gla.sn.pog, Ls-Esth, Li-Esth

Research has extensively examined the role of intra-oral appliances in managing Class III malocclusion during growth. The removable mandibular retractor (RMR) was evaluated in three studies [2,11,34], the bone-anchored intermaxillary traction (BAIMT) in one study [11], the reverse twin block (RTB) in two studies [19,35], the modified Tandem appliance (MTA) in only one study [18], Frankel's function regulator III in only one study (FR-III) [36], and the orthodontic removable traction appliance (ORTA) [6], which can also be called lower-clear-plate based intermaxillary traction (LCP-IMT).

Three studies evaluated the effectiveness of the RMR. One of these studies compared this appliance with another miniscrew-assisted method called BAIMT [11], while the second study compared the RMR with a modified version of it [34]. The third study compared the RMR with an untreated control group [2]. Two studies investigated the efficacy of the RTB. One study compared the RTB against the facemask, including a non-treated control group for comparison [35]. In the second study, the RTB group was compared with a similar group of patients who received photobiomodulation therapy [19]. One study assessed the role of Frankel’s function regulator in managing Class III malocclusion and compared it with an untreated control group [36]. Whereas, the remaining two studies evaluated the effectiveness of the modified tandem against the facemask [18] and the LCP-IMT appliance against an untreated control group [6].

The active treatment duration (T1-T2) was reported in seven studies and varied from 4.3 months to 1.9 years [2,6,11,18,34-36]. One study reported an overall treatment duration, including a retention phase following the active treatment phase (T1-T3), of 12 months [11]. Seven of the included studies used lateral cephalometric and cone beam computed tomography (CBCT)images to evaluate the SNA◦, SNB◦, and ANB◦ angles and mandibular length, and assessed changes in these parameters after treatment of skeletal Class III malocclusion in growing patients, using condylion-gnathion (Co-Gn), Wits appraisal, and overjet changes. None of the studies evaluated the changes in study models or extra-oral clinical photos.

Risk of Bias of the Involved Studies

**Figure 3:**
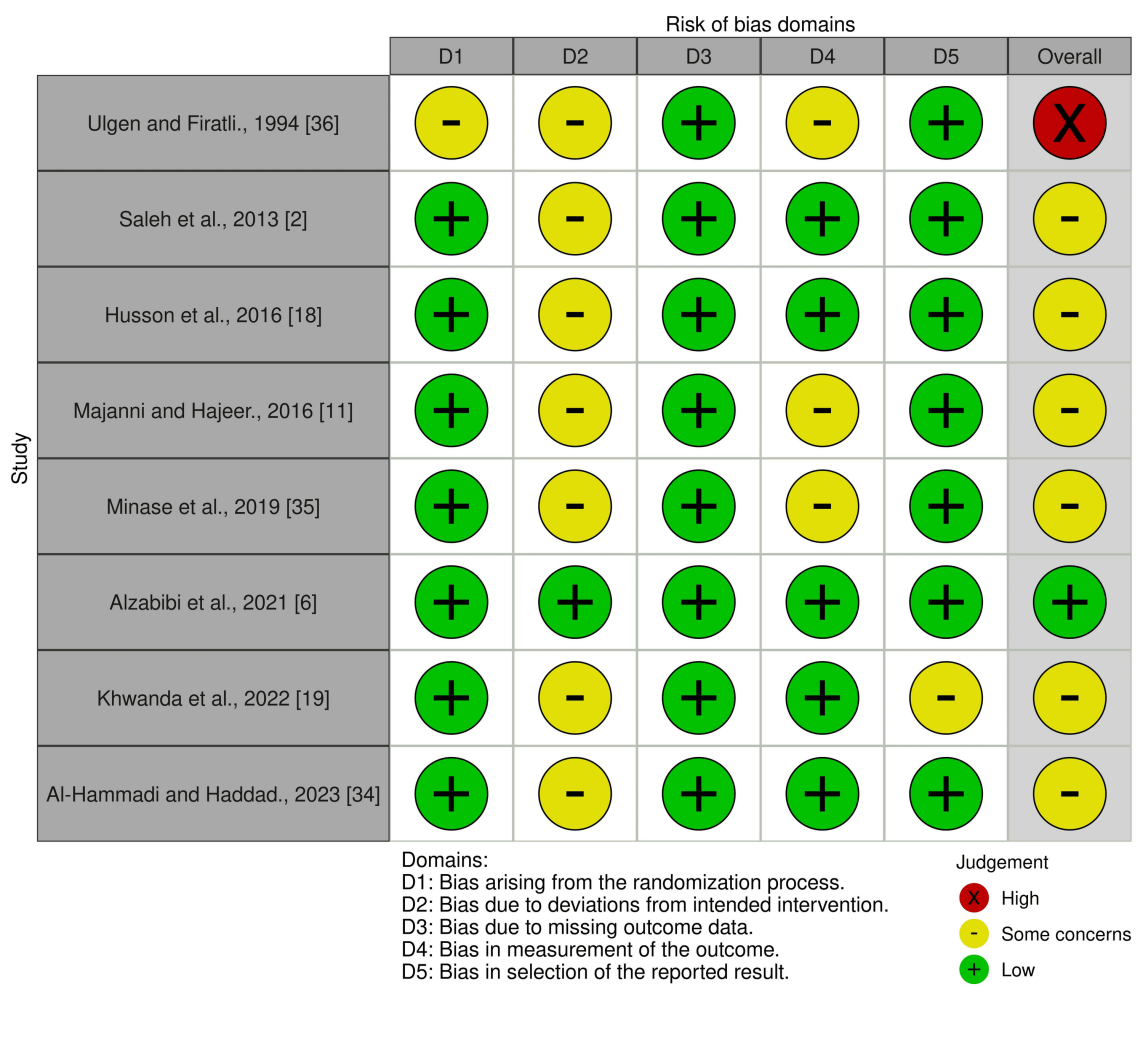
Risk of bias summary - evaluation of each risk of bias domain for the included studies as determined by the review authors

**Figure 4:**
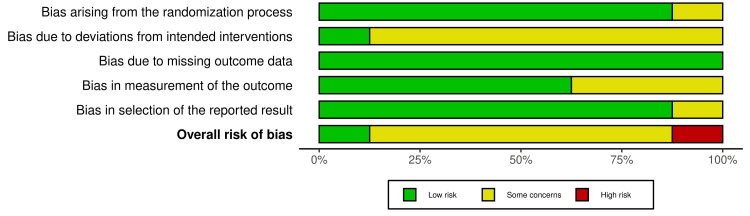
Risk of bias graph: The percentages representing the review authors' assessments of each risk of bias domain across all included studies

Figure 3 presents the bias evaluation for the included studies, and Figure 4 shows the overall bias across domains. One study was judged to be at “high risk of bias” [36], while six studies were assessed as having "some concerns" [2,11,18,19,34,35]. One study was assessed as “low risk of bias” [6]. The high risk of bias in one study is due to (1) inadequate handling of the randomization process, (2) deviation from the intended intervention, and (3) failure in blinding the outcome assessor. Additional details regarding the evaluation and supporting reasons can be found in Appendix 3.

Effects of Intervention

**Figure 5:**
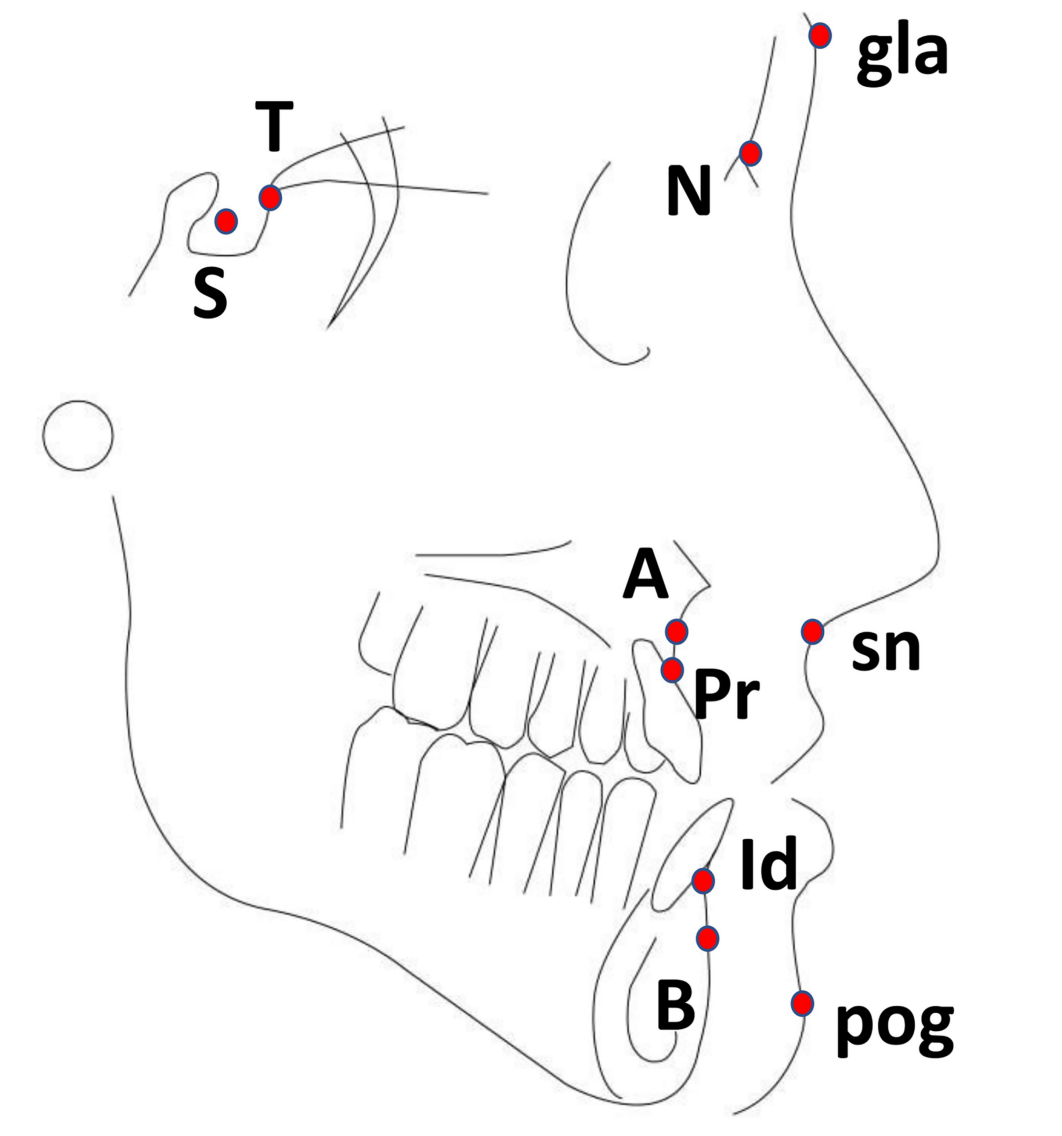
Cephalometric tracing illustrating the landmarks used in different studies. Image created by authors using Adobe Illustrator 2023 (Adobe Inc., San Jose, United States) and Microsoft® PowerPoint® (Microsoft Corporation, Redmond, USA). T: highest point on the front wall of the sella turcica where it meets the tuberculum sella, S (Sella): midpoint center of the sella turcica, N (Nasion): most anterior point on the frontonasal suture, gla (Glabella): most prominent midpoint between the eyebrows (soft-tissue landmark), A (Point A): deepest point on the concavity of the anterior maxillary alveolar ridge, Pr (Prosthion): most inferior and anterior point on the alveolar bone of the maxilla, Id (Infradentale): most superior and anterior point on the alveolar bone of the mandible, sn (Subnasale): point at which the nasal septum meets the upper lip at the base of the columella (soft-tissue landmark), B (Point B): deepest point on the concavity of the anterior mandibular alveolar ridge, pog (Pogonion): most anterior point on the soft-tissue chin, NA plane: cephalometric reference line established from Nasion to Point A, NB plane: cephalometric reference line established from Nasion to Point B.

There were no randomized controlled clinical trials investigating the effects of the Bionator III or Activator III appliances on the early correction of Class III malocclusion in growing patients. Tables 3, 4 present the main results from the included studies. The cephalometric landmarks employed in different studies are illustrated in Figure 5.

**Table 3:** The main findings of the included studies regarding the skeletal changes M-RMR: modified removable mandibular retractor, RMR: removable mandibular retractor, FR: Frankel, BAIMT: bone anchored intermaxillary traction, RTB: reversed twin block, PBMT: photobiomodulation therapy, RTBLP: reverse twin block with lip pads, FM: face mask, MTA: modified tandem appliance, ORTA: orthodontic removable traction appliance, SNA: The angle between the anterior cranial base and NA plane, SNB: The angle between the anterior cranial base and NB plane, ANB: SNA minus SNB, Co: condylion, Gn: gnathion, TV: T Vertical, Wits: distance between AO-BO, A-TV: horizontal distance between point A and the T vertical line, B-TV: horizontal distance between point B and the T vertical line, AO-BO: distance measured between perpendicular projections of Point A (AO) and Point B (BO) onto the occlusal plane.

Authors and Year of Publication	Groups	Skeletal changes						
		SNA	SNB	ANB	Co-Gn	Point A-TV	Point B-TV	Wits
Ulgen and Firatli, 1994 [36]	FR III	0.5°	-0.7°	1.3°				
	Control	0.3°	0.7°	-0.4°				
Saleh et al., 2013 [2]	RMR					1.87 mm	0.17 mm	
	Control					0.40 mm	2.04 mm	
Husson et al., 2016 [18]	MTA	1.38°	-0.44°	1.88°	1.13 mm	1.31 mm		
	FM	1.5°	-0.69°	2.13°	1.25 mm	1.56 mm		
Majanni and Hajeer, 2016 [11]	RMR	0.65°	-0.99°	1.64°	3.35 mm			
	BAIMT	1.48 °	1.94 °	3.41°	3.57 mm			
Minase et al., 2019 [35]	RTBLP	2°	-1.08°	3.08°	4.38 mm	3.04 mm		4.23 mm
	FM	1.31°	-0.73°	2.04°	5.85 mm	2.50 mm		2.38 mm
	Control	0.81°	1.38°	-0.58°	3.62 mm	1.73 mm		-1.04 mm
Alzabibi et al., 2021 [6]	ORTA	1.31°	-1.85°	3.12°	0.58 mm			5.24 mm
	Control	0.29°	0.97°	-0.69°	1.61 mm			-0.72 mm
Khwanda et al., 2022 [19]	RTB	1.54°	0.88°	2.43°				
	RTB+PBMT	1.93°	-1°	2.93°				
Al Hammadi and Haddad, 2023 [34]	RMR	1.63°	-0.57°	2.18°	4.34 mm	2.08 mm		
	M-RMR	2.25°	-0.64°	2.38°	4.35 mm	3.32 mm		

**Table 4:** The main findings of the included studies regarding dentoalveolar and soft-tissue changes M-RMR: modified removable mandibular retractor, RMR: removable mandibular retractor, FR: Frankel, BAIMT: bone-anchored intermaxillary traction, RTBLP: reverse twin block with lip pads, FM: face mask, MTA: modified tandem appliance, ORTA: orthodontic removable traction appliance, U1-SN: The angle between the anterior cranial base and the upper incisor axis, L1-Go-Me: The angle between the mandibular plane and the lower incisor axis, Pr: prosthion, Id: infradental, gla.sn.pog: The facial convexity angle, Ls-Esth: The distance between the Labrale superius and E-Line of Ricketts, Li-Esth: The distance between the Labrale inferius and E-Line of Ricketts, NasoLab: The nasolabial angle, MentoLab: The mentolabial angle, TV: T Vertical

Authors	Groups	Dentoalveolar changes						Soft-tissue changes				
		U1-SN	L1-Go-Me	Overjet	Overbite	Id-TV	Pr-TV	Nasolab	Mentolab	gla.sn.pog	Ls-Esth	Li-Esth
Ulgen and Firatli, 1994 [36]	FR III	4.8°	-5.3°	3.8 mm	-2.1 mm							
	Control	2.6°	1.1°	-0.3 mm	0.3 mm							
Saleh et al., 2013 [2]	RMR					0.35 mm	1.92 mm	-8.87°	5.24°	5.13°		
	Control					1.89 mm	0.50 mm	2.16°	0.58°	0.11°		
Husson et al., 2016 [18]	MTA			2.25 mm	-0.4 mm							
	FM			3.53 mm	-1.76 mm							
Majanni and Hajeer, 2016 [11]	RMR	5.22°	-2.55°	4.20 mm	-1.91 mm			-3.72°	4.90°	-4.81°	0.45 mm	-0.34 mm
	BAIMT	2.71°	0.88°	4.90 mm	-1.80 mm			-5.64°	8.59°	-7.11°	1.41 mm	-0.53 mm
Minase et al., 2019 [35]	RTBLP	3.08°	0.23°									
	FM	5.46°	-0.62°									
	Control	2.38°	-2.62°									
Alzabibi et al., 2021 [6]	ORTA	2.96°	-7.38°	5.87 mm	0.62 mm			1.12°	−3.45°		1.03 mm	-0.80 mm
	Control	0.50 °	-0.32°	-0.33 mm	0.21 mm			-0.84°	1.79°		-0.74 mm	- 0.20 mm
Al Hammadi and Haddad, 2023 [34]	RMR	4.23°	-5.12°	3.95 mm		-1.25 mm	2.24 mm	-3.50°		4.26 °	2.85 mm	-0.48 mm
	M-RMR	3.36°	-6.28°	5.18 mm		-1.01 mm	3.42 mm	-4.59°		4.91°	2.47 mm	-0.80 mm

Skeletal Changes: Regarding the ANB angle, seven studies assessed the sagittal relationships between the two jaws. Alzabibi et al. reported the greatest improvement with the LCP-IMT appliance, resulting in a mean increase of 3.12° in the ANB angle [6]. The studies by Khwanda et al. and Minase et al. reported that the RTB appliance resulted in mean increases of 3.08° and 2.43°, respectively [19,35]. When the RMR was used, Al-Hammadi and Haddad observed a statistically significant increase in the ANB angle by a mean of 2.18° [34]. Majanni and Hajeer, who also used the same appliance but on older patients (i.e., between 9.5 and 13 years of age), reported a statistically significant increase in the RMR group by a mean of 1.48° [11]. Similarly, Husson et al. reported a statistically significant increase in the ANB angle, with a mean change of 1.88° using the modified tandem appliance [18]. Ulgen et al. compared the Frankel III regulator with an untreated control group and reported the least improvement in the ANB angle, with a mean increase of 1.3° [36].

Regarding Wits appraisal, Alzabibi et al. reported that the LCP-IMT group showed a significant increase in Wits appraisal compared with the untreated control group, with a mean increase of 5.24 mm [6]. Additionally, Minase et al. reported that the RTB group experienced a mean increase of 4.23 mm in Wis appraisal compared to the untreated control group [35].

When evaluating the maxillary position using the SNA angle, Minase et al reported the most significant increase in their study when they used the RTB appliance (a mean of 2°) [35]. Khwanda et al., who also used the RTB, observed a mean increase in the SNA angle of 1.54° [19]. When the RMR was used, Al-Hammadi and Haddad reported a mean increase of 1.63° [34]. On the other hand, Majanni and Hajeer also used the RMR and reported a smaller increase in the SNA angle (0.65°) [11] compared with the previous study; this may be due to the older age of the included patients in the Majanni and Hajeer study. The MTA appliance showed an increase in the SNA angle by a mean of 1.38° [18], which was similar to the LCP-IMT appliance (1.31°) [6]. The least improvement was observed in Ulgen et al.'s study, which used the Frankel III regulator and reported a mean increase of 0.5° [36].

When assessing the sagittal position of the mandible using the SNB angle, Alzabibi et al. reported the greatest decrease in the SNB angle with the LCP-IMT appliance, with an average decrease of 1.85° [6]. The remaining five studies showed a similar decrease in the SNB angle with an average range from 0.44° to 1.08° [18,19,34-36].

When assessing the maxillary sagittal position through the evaluation of point A position in reference to the “T vertical line”, Minase et al., who compared the RTB and the face mask in the presence of the control group, reported the most statistically significant increase in the RTB group by a mean of 3.04 mm [35]. Husson et al., who compared the modified tandem appliance to the face mask, also showed a statistically significant increase of 1.31 mm [18].

**Figure 6:**
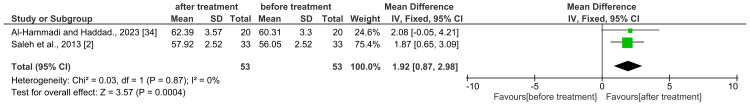
Forest plot of the change of Point A between “before” and “after” treatment

A quantitative synthesis of the data was possible for the maxillary sagittal position assessed using Point A, as shown in Figure 6. Two studies were included in which the RMR treatment was applied [2,34]. Point A moved forward by a mean of 1.92 mm following active treatment, a statistically significant change with low heterogeneity among studies (MD = 1.92; 95% CI: 0.87, 2.98; p = 0.0004; χ² = 0.03; p = 0.87; I² = 0%). GRADE assessment (Table 5) indicated that the quality of evidence for this outcome is low.

**Table 5:** Summary of findings table according to the Grading of Recommendations, Assessment, Development, and Evaluation guidelines for the included trials High quality: Further research is very unlikely to change our confidence in the estimate of effect, Moderate quality: Further research is likely to have an important impact on our confidence in the estimate of effect and may change the estimate, Low quality: Further research is very likely to have an important impact on our confidence in the estimate of effect and is likely to change the estimate, Very low quality: we are very uncertain about the estimate. Grading of Recommendations, Assessment, Development, and Evaluation (GRADE) guidelines [33]. ^a^Decline one level for risk of bias (some concerns due to deviation from intended intervention), and one level for imprecision, ^b^Decline one level for risk of bias (some concerns due to deviation from intended intervention), and one level for imprecision, ^c^Decline one level for risk of bias (some concerns due to deviation from intended intervention), and one level for imprecision, ^d^Decline one level for risk of bias (some concerns due to deviation from intended intervention), and one level for imprecision, ^e^Decline one level for risk of bias (some concerns due to deviation from intended intervention), and one level for imprecision, ​​​​​​​^f^Decline one level for risk of bias (some concerns due to deviation from intended intervention), one level for Inconsistency and one level for imprecision CI: confidence interval, RCT: randomized controlled clinical trial, TV: T Vertical, Pr: prosthion, Id: infradental, gla.sn.pog: the facial convexity angle, A-TV: horizontal distance between point A and the T vertical line, B-TV: horizontal distance between point B and the T vertical line.

Quality assessment criteria						Summary of findings			Comments
No. of studies	Risk of bias	Inconsistency	Indirectness	Imprecision	Other considerations	No. of patients	Effects	Certainty	
							Relative (95% CI)		
Point A-TV change									
2 RCTs	Serious	Not serious	Not serious	Serious	None	106	Mean 1.92 mm CI 95% (0.87,2.98)	⊕⊕OO^a^ Low	The difference between the changes before and after treatment was statistically significant. With a low quality of evidence. ⊕⊕OO^a^
Point B-TV change									
2 RCTs	Serious	Not serious	Not serious	Serious	None	106	Mean 0.13 mm CI 95% (-0.98, 1.25)	⊕⊕OO^b^ Low	The difference between the changes before and after treatment was not statistically significant. With a low quality of evidence. ⊕⊕OO^b^
gla.sn.pog change									
2 RCTs	Serious	Not serious	Not serious	Serious	None	106	Mean 4.92° CI 95% (3.51, 6.32)	⊕⊕OO^c^ Low	The difference between the changes before and after treatment was statistically significant. With a low quality of evidence. ⊕⊕OO^c^
Point Pr-TV change									
2 RCTs	Serious	Not serious	Not serious	Serious	None	106	Mean 1.97 mm CI 95% (0.87,3.07)	⊕⊕OO^d^ Low	The difference between the changes before and after treatment was statistically significant. With a low quality of evidence. ⊕⊕OO^d^
Point Id-TV change									
2 RCTs	Serious	Not serious	Not Serious	Serious	None	106	Mean 0.16 mm CI 95% (-0.85,1.17)	⊕⊕OO^e^ low	The difference between the changes before and after treatment was not statistically significant. With a low quality of evidence. ⊕⊕OO^e^
Nasolabial angle change									
2 RCTs	Serious	Serious	Not Serious	Serious	None	106	Mean -5.18 CI 95% (-7.54, -2.83)	⊕OOO^f^ Very low	The difference between the changes before and after treatment was statistically significant. With a very low quality of evidence. ⊕OOO^f^

**Figure 7:**
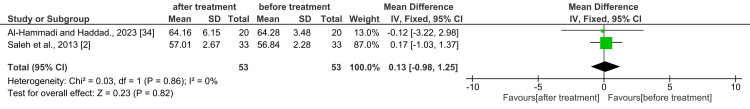
Forest plot of the changes of Point B between “before” and “after” treatment

Quantitative data analysis was also conducted to assess mandibular sagittal position, measured by the position of point B, as illustrated in Figure 7. Two studies were included in this analysis, both of which applied RMR treatment [2,34]. After the end of active treatment, point B moved forward by a mean of 0.13 mm. This change was not statistically significant, with low heterogeneity among the studies (MD = 0.13; 95% CI: -0.98, 1.25; p = 0.82; χ² = 0.03; p = 0.86; I² = 0%; Figure 7). GRADE assessment indicated that the quality of evidence for this outcome is low. 

When assessing the length of the mandible (Co-Gn), Alzabibi et al. utilized the LCP-IMT appliance and observed the least increase in mandibular length, with a mean of 0.58 mm [6], followed by Husson et al., who used the MTA appliance and observed an increase by a mean of 1.13 mm [18]. Meanwhile, Majanni and Hajeer observed an increase in the RMR group with a mean of 3.35 mm [11]. The largest increases in mandibular length were observed in the studies by Minase et al. and Al-Hammadi and Haddad, with mean increases of 4.38 mm and 4.34 mm, respectively [34,35].

Dentoalveolar Changes: When assessing overjet changes, Alzabibi et al. reported the most significant improvement with the LCP-IMT appliance, with a mean increase of 5.87 mm [6]. When the RMR was used, both Majanni and Hajeer and Al-Hammadi and Haddad reported similar overjet corrections, with mean increases of 4.20 mm and 3.95 mm, respectively [11,34]. Ulgen et al. observed a mean increase of 3.8 mm in the overjet with the Frankel III appliance [36]. The least improvement was observed in the study by Husson et al., which reported a mean increase of only 2.25 mm [18].

Regarding changes in overbite, two studies (using the RMR and FR-III appliances) reported an average decrease following active treatment, with mean decreases of 1.91 mm and 2.1 mm, respectively [11,36]. Husson et al. observed a smaller decrease of 0.4 mm in the overbite with the modified tandem appliance [18]. In contrast, Alzabibi et al. reported a mean increase of 0.62 mm with the LCP-IMT appliance [6].

**Figure 8:**
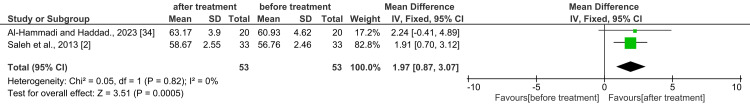
Forest plot of the changes of Point Pr between “before” and “after” treatment

A quantitative data analysis was conducted to assess the maxillary dentoalveolar position, measured by the position of the point Prosthion (Pr), as illustrated in Figure 8. Two studies were included in this meta-analysis, both of which used the RMR appliance [2,34]. After the active treatment, the position of point Pr moved forward by a mean of 1.97 mm. This change was statistically significant and showed low heterogeneity between the studies (MD = 1.97; 95% CI: 0.87, 3.07; p = 0.0005; χ² = 0.05; p = 0.82; I2 = 0%). Using the GRADE guidelines, the evidence supporting this outcome was deemed low.

**Figure 9:**
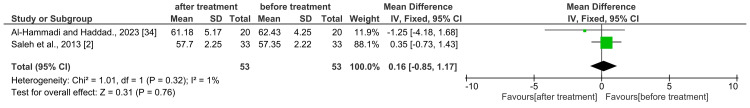
Forest plot of the changes of Point Id between “before” and “after” treatment

The mandibular dentoalveolar position was quantitatively assessed through the infradental point (Id) position, as shown in Figure 9. Two studies were included in this analysis, both of which utilized the RMR appliance [2,34]. Following active treatment, point Id advanced by an average of 0.16 mm. This change showed low heterogeneity across the studies and was not statistically significant (MD = 0.16; 95% CI: -0.85, 1.17; p = 0.76; χ² = 1.01; p = 0.32; I² = 1%). The quality of the evidence regarding this outcome was low, according to GRADE.

When analyzing the upper incisors’ inclination in relation to the cranial base (U1-SN), five studies reported an increase in upper incisor inclination following active treatment, with mean changes ranging from 2.71° to 5.22° [6,11,34-36].

Regarding lower incisor inclination, Majanni and Hajeer found that the inclination of the lower incisor in reference to the mandibular plane (L1-GoMe) decreased when using the RMR appliance by an average of 2.55° [11]. Three other studies reported similar decreases in lower incisor inclination following active treatment, with a mean change ranging from 5.12° to 7.38° [6,34,36].

**Figure 10:**
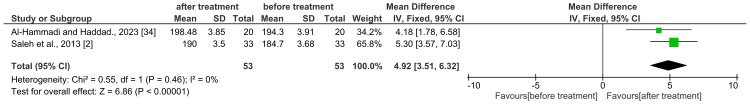
Forest plot of the change in the facial convexity angle between “before” and “after” treatment

Soft-Tissue Changes: Three studies assessed facial convexity using gla.sn.pog angle. According to Majanni and Hajeer, the facial convexity angle increased by an average of 4.81° with the RMR appliance [11]. A quantitative data synthesis of the remaining two studies [2,34], shown in Figure 10, indicated that the RMR treatment led to a statistically significant increase in the gla.sn.pog angle by a mean of 4.92°, with low heterogeneity between the studies. (MD = 4.92; 95% CI: 3.51, 6.32; p = 0.00001; χ² = 0.55; p = 0.46; I2 = 0%). GRADE assessment indicated that the quality of evidence for this outcome is low.

**Figure 11:**
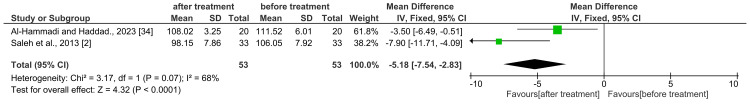
Forest plot of the change in the nasolabial angle between “before” and “after” treatment

Four studies evaluated the nasolabial angle after active treatment. Two studies were included in the quantitative analysis of the RMR appliance [2,34], as shown in Figure 11. The nasolabial angle decreased by an average of 5.18°, a statistically significant difference despite high heterogeneity across studies. (MD = -5.18; 95% CI: -7.54, -2.83; p = 0.0001; χ² = 3.17; p = 0.07; I2 = 68%). GRADE assessment indicated that the quality of evidence for this outcome is very low. Majanni and Hajeer reported a mean decrease in the nasolabial angle of 3.72° in the RMR group [11]. In contrast, Alzabibi et al. reported a mean increase in the nasolabial angle by 1.12° using the LCP-IMT appliance [6].

Regarding the mentolabial angle, Saleh et al. and Majanni and Hajeer both reported similar increases in the mentolabial angle using the RMR, with a mean increase of 5.24° and 4.90°, respectively [2,11]. However, Alzabibi et al. reported a mean decrease of 3.45° when using the LCP-IMT appliance [6].

Three studies investigated the distance separating the Labrale Superius from the E-Line of Ricketts, reporting average increases ranging from 0.45 mm to 2.85 mm [6,11,34]. In the same studies, the distance between the Labrale Inferius and the E-Line showed a mean decrease that ranged from 0.34 mm to 0.80 mm.

Discussion

Although extra-oral appliances can effectively address skeletal Class III malocclusion, their success is often limited by poor patient cooperation [18]. Their bulky design and unappealing appearance frequently reduce willingness to wear them, making consistent use difficult. As a result, achieving optimal outcomes with these appliances requires a high level of motivation. In contrast, intra-oral non-skeletally anchored appliances are typically better accepted by patients and tend to show improved compliance [19].

This is, to the best of our knowledge, the first systematic review assessing RCT-based evidence on hard- and soft-tissue changes following treatment of skeletal Class III malocclusion in growing patients using non-skeletally anchored intra-oral appliances. However, the studies included in the review varied significantly in interventions, age groups, and assessment methods, resulting in only two of eight articles being suitable for meta-analysis, which limits the ability to draw strong conclusions about the magnitude of hard- and soft-tissue changes.

There is a consensus among the included studies [6,11,18,19,34-36] that the sagittal relationship between the two jaws improved after treatment, most notably evidenced by an increase in the ANB angle across all appliances, with the LCP-IMT reporting the most significant increase (MD: 3.12°) [6]. This enhancement is attributed to the appliance's design, which combines intraoral elastic mechanics with rapid maxillary expansion. However, three-fourths of this improvement was primarily attributed to mandibular skeletal changes, indicating a significant clockwise rotation of the mandible, which may not be suitable for patients with long faces. This rotational pattern is clinically relevant, as clockwise rotation is associated with increased lower anterior facial height and a downward-backward displacement of the chin. While this may be beneficial in patients with reduced vertical proportions or deep bite tendencies, it is contraindicated in individuals with hyperdivergent growth patterns (“long face” phenotype). In such patients, further clockwise rotation can exacerbate vertical disproportions, increase anterior facial height, worsen lip incompetence, and reduce chin projection, ultimately compromising facial aesthetics and stability. The Wits appraisal further supported sagittal correction in cases treated with RTB and LCP-IMT [6,35], showing an increase in the linear distance, which reinforced the angular changes observed in the ANB angle.

In terms of maxillary changes, all studies reported an increase in the SNA angle [6,11,18,19,34-36], with the modified RMR appliance reporting the greatest improvement (MD: 2.25°), likely due to the use of upper lip pads that provide forward traction via soft-tissue stimulation [34]. Complementing this angular enhancement, the meta-analysis findings confirmed a statistically significant anterior movement of point A relative to the T vertical reference line when using the standard RMR (MD: 1.92 mm) [2,34], despite the supporting evidence being graded as low quality according to GRADE. Notably, Minase et al. reported the greatest forward movement of point A (MD: 3.04 mm) [35], using a combination of RTB, upper lip pads, and rapid maxillary expansion, suggesting a synergistic effect among these elements in promoting maxillary advancement.

Mandibular skeletal outcomes were also consistent, as all studies reported a decrease in the SNB angle following treatment [6,11,18,19,34-36], with the largest reduction observed in Alzabibi et al.'s study using the LCP-IMT appliance (MD: 1.85°) [6], reinforcing the pattern of clockwise mandibular rotation. However, the meta-analysis revealed no significant change in point B position after active treatment with the RMR appliance (MD: 0.13 mm) [2,34], with moderate evidence according to GRADE. This result may be due to a significant reduction in the gonial angle, resulting from the condyle's upward-forward growth direction. As a result, the RMR treatment did not limit the growth of the mandible but modified its shape through an anterior morphogenetic rotation. Supporting this result, all studies evaluating mandibular length (Co-Gn) found post-treatment increases [6,11,18,34,35], though the extent varied; LCP-IMT demonstrated the smallest increase in mandibular length (MD: 0.58 mm) [6], whereas RTB showed the largest increase (MD: 4.38 mm) [35], which is likely due to the RTB primarily affecting the maxilla and having a limited impact of restriction on mandibular growth.

Dentoalveolar adjustments were strongly correlated with these skeletal changes. Overjet increased consistently following treatment with RMR, LCP-IMT, MTA, and FR-III appliances [6,11,18,34,36], driven by both improvements in the sagittal relationship between the two jaws and changes in the inclination of the upper and lower incisors. Overbite changes were more variable: while three studies reported reductions associated with clockwise mandibular rotation [11,18,36], Alzabibi et al. noted an increase [6], potentially due to the use of Class III elastics, which may promote lower incisor eruption and vertical anterior overlap. In evaluating maxillary dentoalveolar positioning, the RMR appliance produced a statistically significant forward movement of point Prosthion (Pr) (MD: 1.97 mm) [2,34], aligning with the maxillary skeletal advancement, though the supporting evidence remains of low quality according to GRADE. In contrast, when evaluating mandibular dentoalveolar positioning, the point Infradental (Id) showed no statistically significant change with the RMR appliance (MD: 0.16 mm) [2,34], with low evidence supporting this finding. This finding strengthens the underlying assumption that mandibular remodelling occurred via rotation rather than linear movement. It also emphasizes the role of dentoalveolar adaptation in response to skeletal remodelling. Incisors' inclination patterns reinforce this interrelationship: upper incisors showed increased proclination across studies [6,11,34-36], coinciding with forward maxillary movement, while lower incisors exhibited lingual tipping following treatment with RMR and LCP-IMT [6,11,34,36], attributed to the reversed labial bow, which exerts pressure on the cervical margins of the mandibular anterior teeth and the application of Class III elastics in the LCP-IMT appliance.

Soft-tissue changes reflected the underlying skeletal and dental improvements and were critical in defining the facial profile. The meta-analysis results showed an improvement in facial convexity after RMR treatment, demonstrated by increased gla.sn.pog angle values (MD: 4.92°) [2,34], supported by similar results from Majanni and Hajeer (MD: 4.81°) [11], indicating a more harmonious facial profile following the sagittal relationship correction between the two jaws. The nasolabial angle showed a notable reduction after active treatment with the RMR appliance (MD: 5.18°) [2,34], likely due to upper incisor proclination and the forward movement of point Prosthion, though the evidence supporting this outcome was classified as very low according to GRADE. An increase in the mentolabial angle was reported in two studies [2,11] and was attributed to reduced incisor retroclination, which allowed improved soft-tissue adaptation of the lower lip. Regarding upper-lip changes, three studies in this review reported that the distance between the upper-lip landmark, Labrale Superius, and Ricketts E-Lines significantly increased after active treatment with the RMR, M-RMR, and LCP-IMT appliances [6,11,34]. This change is attributed to the forward movement of the maxilla and increased upper incisor inclination. However, the same three studies observed a notable decrease in the length between the lower lip (Labrale Inferius) and Ricketts’ E-Line after active treatment with the same appliances. This change is due to backward movement of the mandible and decreased lower incisor inclination, resulting in a more retruded lower lip position and a compatible lip profile.

These findings collectively demonstrate the effects of intra-oral, non-skeletally anchored appliances on skeletal, dental, and soft-tissue structures. The combination of mandibular rotation and maxillary advancement, along with dentoalveolar adaptations, results in meaningful changes to facial aesthetics. The overall impact varies by appliance design, and it should be selected carefully based on each patient’s needs.

Limitations

Important variables, such as the vertical skeletal measurements, were not included in the data covered by this review, as the focus was primarily on the sagittal skeletal variables. Furthermore, only one of the eight included studies had a low risk of bias, which reduced trust in the validity of the results. Overall, the quality of the evidence ranged from low to very low. Therefore, high-quality RCTs are required to accurately evaluate hard- and soft-tissue changes following Class III malocclusion treatment with intra-oral, non-skeletally anchored appliances. The high degree of heterogeneity and the variety of intra-oral appliances used hindered the inclusion of all studies in the meta-analysis, resulting in data being pooled from only two studies. Given the different methods available for correcting Class III malocclusion, a network meta-analysis might be the most reliable approach to compare the effectiveness of the various appliances included in this review.

## Conclusions

Findings suggest intra-oral non-skeletally anchored appliances positively affect hard and soft tissues, with LCP-IMT and RTB showing the greatest sagittal improvement, followed by M-RMR, RMR, MTA, and FR-III. LCP-IMT most effectively limited mandibular growth but caused unfavorable lower incisor retroclination, increasing relapse risk, while RTB and RMR had minimal impact on mandibular length. LCP-IMT also produced the greatest overjet and was the only device to increase overbite. RMR advanced dentoalveolar points (Pr, Id), and soft tissue changes were similar across devices, improving facial convexity and lip position. However, studies were limited by short treatment durations (4-9 months) and a lack of long-term follow-up data. Evidence quality ranged from low to very low, restricting strong conclusions.

## Appendices

Appendix 1

**Table 6:** The electronic search strategy for each database

Electronic database	Search strategy
CENTRAL (The Cochrane Library)	#1 "Class III malocclusion " OR " skeletal Class III" OR "mesial occlusion" OR "mandibular prognathism" #2 "growth modification" OR "Class III skeletal anchorage" OR "functional treatment" OR "functional orthopedic" OR "jaw relationship correction" OR "maxillary advancement" OR "maxillary protrusion" OR "mandibular restriction" OR "maxillary protrusion appliance " OR "removable functional appliance" OR "Frankel regulator III" OR "Bionator III" OR "Activator III" OR "modified tandem appliance" OR "removable mandibular retractor" OR "reverse twin block" #3 "soft tissue " OR "soft-tissue " OR" lip profile " OR " Facial profile "OR " Ricketts line" OR "E-line" OR "facial convexity" OR "nasolabial angle " OR "mentolabial angle " OR "upper lip position" OR "lower lip position" #1 AND #2 AND #3
PubMed	#1 "Class III malocclusion " OR " skeletal Class III" OR "mesial occlusion" OR "mandibular prognathism" #2 "growth modification" OR "Class III skeletal anchorage" OR "functional treatment" OR "functional orthopedic" OR "jaw relationship correction" OR "maxillary advancement" OR "maxillary protrusion" OR "mandibular restriction" OR "maxillary protrusion appliance " OR "removable functional appliance" OR "Frankel regulator III" OR "Bionator III" OR "Activator III" OR "modified tandem appliance" OR "removable mandibular retractor" OR "reverse twin block" #3 "soft tissue " OR "soft-tissue " OR" lip profile " OR " Facial profile "OR " Ricketts line" OR "E-line" OR "facial convexity" OR "nasolabial angle " OR "mentolabial angle " OR "upper lip position" OR "lower lip position" #1 AND #2 AND #3
Scopus	#1TITLE-ABS-KEY ("Class III malocclusion " OR " skeletal Class III" OR "mesial occlusion" OR "mandibular prognathism") #2TITLE-ABS-KEY ("growth modification" OR "Class III skeletal anchorage" OR "functional treatment" OR "functional orthopedic" OR "jaw relationship correction" OR "maxillary advancement" OR "maxillary protrusion" OR "mandibular restriction" OR "maxillary protrusion appliance " OR "removable functional appliance" OR "Frankel regulator III" OR "Bionator III" OR "Activator III" OR "modified tandem appliance" OR "removable mandibular retractor" OR "reverse twin block") #3TITLE-ABS-KEY ("soft tissue " OR "soft-tissue " OR" lip profile " OR " Facial profile "OR " Ricketts line" OR "E-line" OR "facial convexity" OR "nasolabial angle " OR "mentolabial angle " OR "upper lip position" OR "lower lip position") #1 AND #2 AND #3
Web of Science	#1TS= ("Class III malocclusion " OR " skeletal Class III" OR "mesial occlusion" OR "mandibular prognathism") #2TS= ("growth modification" OR "Class III skeletal anchorage" OR "functional treatment" OR "functional orthopedic" OR "jaw relationship correction" OR "maxillary advancement" OR "maxillary protrusion" OR "mandibular restriction" OR "maxillary protrusion appliance " OR "removable functional appliance" OR "Frankel regulator III" OR "Bionator III" OR "Activator III" OR "modified tandem appliance" OR "removable mandibular retractor" OR "reverse twin block") #3TS= ("soft tissue " OR "soft-tissue " OR" lip profile " OR " Facial profile "OR " Ricketts line" OR "E-line" OR "facial convexity" OR "nasolabial angle " OR "mentolabial angle " OR "upper lip position" OR "lower lip position") #1 AND #2 AND #3
Google Scholar	(Class III malocclusion OR skeletal Class III OR mesial occlusion OR mandibular prognathism) AND (growth modification OR Class III skeletal anchorage OR functional treatment OR functional orthopedic OR jaw relationship correction OR maxillary advancement OR maxillary protrusion OR mandibular restriction OR maxillary protrusion appliance OR removable functional appliance OR Frankel regulator III OR Bionator III OR Activator III OR reverse Twin Block OR removable mandibular retractor OR modified tandem appliance) AND (soft tissue OR soft-tissue OR lip profile OR Facial profile OR Ricketts line OR E-line OR "facial convexity OR nasolabial angle OR mentolabial angle OR upper lip position OR lower lip position )
World Health Organization (WHO) International Clinical Trials Registry Platform (ICTRP)	(functional orthodontic treatment OR growth modification OR Class III skeletal anchorage OR maxillary advancement OR functional appliance) AND (hard tissue changes OR soft tissue changes)
ClinicalTrials.gov	(functional orthodontic treatment OR growth modification OR Class III skeletal anchorage OR maxillary advancement OR functional appliance) AND (hard tissue changes OR soft tissue changes)

Appendix 2

**Table 7:** Excluded studies and the reasons beyond exclusion

Excluded study	Reason for exclusion
Galeotti A, Martina S, Viarani V, Franchi L, Rongo R, D'Antò V, Festa P. Cephalometric effects of Pushing Splints 3 compared with rapid maxillary expansion and facemask therapy in Class III malocclusion children: a randomized controlled trial. Eur J Orthod. 2021 Jun 8;43(3):274-282. doi: 10.1093/ejo/cjaa076.	The patients included were under 6 years old.
Garattini, G., et al. (1998). "Skeletal and dental modifications produced by the Bionator III appliance." American Journal of Orthodontics and Dentofacial Orthopedics 114(1): 40-44.	Retrospective study.

Appendix 3

**Table 8:** Details of the risk of bias assessment of the studies using the Cochrane's Risk of Bias tool 2.0

Study	Bias arising from the randomization process	Bias due to deviations from intended interventions	Bias due to missing outcome data	Bias in the measurement of the outcome	Bias in the selection of the reported result	Over-all bias
	D1	D2	D3	D4	D5	
Ulgen and Firatli., 1994 [36]	some concerns: The method used for randomization was not reported. “The patients were divided randomly into treatment and control groups”	Some concerns: No details about blinding, either the patient or clinician.	Low risk: No dropouts were reported.	Some concerns: No details of the blinding of outcome assessors were reported.	Low risk: No information about the registration protocol was mentioned. However, the reported outcomes in the result section seemed to correspond with the pre-defined outcomes aforesaid in the method section.	High risk
Saleh et al., 2013 [2]	Low risk “The randomization procedure was performed manually by simply asking each participant to pick up a concealed opaque envelope from a black plastic box.”	Some concerns: No details about blinding, either the patient or clinician.	Low risk: “Three patients in the treatment group failed to complete the study because of a lack of compliance and were excluded from analysis. On the other hand, two patients in the control group were excluded from the study because they moved to another country”. Nearly all the outcome data is available (93%).	Low risk: The outcome assessor was blinded.	Low risk: No information about the registration protocol was mentioned. However, the reported outcomes in the result section seemed to correspond with the pre-defined outcomes aforesaid in the method section.	Some concern
Husson et al., 2016 [18]	Low risk: “Patients were randomized in a 1:1 ratio into two equal groups using an online randomization service”	Some concerns: “Blinding of either patient or operator was not possible”. We judge that the outcome might be influenced by the lack of blinding.	Low risk: No dropouts were reported.	Low risk: The outcome assessor was blinded.	Low risk: The protocol for the study was registered in clinical trial.gov study ID: NCT02144324. and the outcomes that mentioned in the protocol have been reported	Some concerns
Majanni and Hajeer., 2016 [11]	Low risk The randomization sequence was computer-generated. The allocation sequence was concealed using sequentially opaque, sealed envelopes.	Some concerns: No details about blinding, either the patient or the clinician.	Low risk: No dropouts were reported.	Some concerns: No details of the blinding of outcome assessors were reported.	Low risk: No information about the registration protocol was mentioned. However, the reported outcomes in the result section seemed to correspond with the pre-defined outcomes aforesaid in the method section.	Some concerns
Minase et al., 2019 [35]	Low risk: “Simple randomization was performed by creating a randomization list using Minitab® V16 with an allocation ratio of 1:1:1.”	Some concerns: No details about blinding, either the patient or the clinician.	Low risk: No dropouts were reported.	Some concerns: No details of the blinding of outcome assessors were reported.	Low risk: No information about the registration protocol was mentioned. However, the reported outcomes in the result section seemed to correspond with the pre-defined outcomes aforesaid in the method section.	Some concern
Alzabibi et al., 2021 [6]	Low risk “The randomization sequence was computer-generated. The allocation sequence was concealed using sequentially opaque, sealed envelopes.”	Low risk: “Due to the nature of the trial, blinding of the patients and the clinicians were not applicable”. We judge that the outcome is not likely to be influenced by the lack of blinding.	Low risk: Two patients were lost to follow-up due to personal reasons. Nearly all the outcome data is available (95%).	Low risk: The outcome assessor was blinded.	Low risk: The protocol for the study was registered in clinical trial.gov study ID: NCT 03172442 and the outcomes mentioned in the protocol have been reported.	Low risk
Khwanda et al., 2022 [19]	Low risk “Patients were assigned to the irradiation group (RTB+PBMT) or the control group (RTB) with an allocation ratio of 1:1 using a simple randomization technique. Each patient was asked to select a folded piece of paper from a box containing 40 pieces of paper”.	Some concerns: “Blinding of patients or the principal researcher was impossible”. We judge that the outcome might be influenced by the lack of blinding.	Low risk: No dropouts were reported.	Low risk: The outcome assessor was blinded.	Some concerns: The protocol for the study was registered on the German Clinical Trials Register (DRKS-ID: DRKS00027535) but the outcomes mentioned in the protocol have not been all reported.	Some concerns
Al Hammadi and Haddad., 2023 [34]	Low risk: “The sample was distributed by (1:1) allocation ratio into both groups using the simple computer random method.”	Some concerns: “Blinding of both the examiner and the patient was not applicable”. We judge that the outcome might be influenced by the lack of blinding.	Low risk: No dropouts were reported.	Low risk: The outcome assessor was blinded.	Low risk: The protocol for the study was registered on the German Clinical Trials database (Identifier: DRKS00027155) and the outcomes mentioned in the protocol have been reported.	Some concern
